# E2F1 promotes cancer cell sensitivity to cisplatin by regulating the cellular DNA damage response through miR-26b in esophageal squamous cell carcinoma

**DOI:** 10.7150/jca.33983

**Published:** 2020-01-01

**Authors:** Kun Zhang, Bo Zhang, Yun Bai, Limeng Dai

**Affiliations:** 1 Department of Pathogenic Biology, College of Basic Medical Science, Army Medical University (Third Military Medical University), Chongqing 400038, PR China;; 2 Department of Medical Genetics, College of Basic Medical Science, Army Medical University (Third Military Medical University), Chongqing 400038, PR China.

**Keywords:** E2F1, miR-26b, ATM, esophageal squamous cell carcinoma, cisplatin.

## Abstract

Cisplatin is an essential chemotherapy drug in esophageal squamous cell carcinoma (ESCC). Some studies suggested that the expression of E2F1 is increased in ESCC cells after cisplatin treatment, but its mechanism remains obscure. Here, we found that miR-26b is upregulated in ESCC cell lines with cisplatin treatment, and it relies on the expression of E2F1 because E2F1 directly binds to the promoter of the miR-26b gene, thus activating the transcriptional activity of miR-26b. Cell cycle analysis suggested that miR-26b inhibits the G1/S phase transition, thus inhibiting the cell growth of ESCC cells. The cisplatin-induced cycle arrest also closely depends on the expression of miR-26b. *In vivo* assays revealed that the sensitivity of ESCC cells to cisplatin is decreased when the E2F1/miR-26b pathway is disturbed. A nude mouse xenograft model of cisplatin treatment showed that the tumor volume was increased in the Si-E2F1 group compared with that in the group with cisplatin treatment alone. The effect may be due to the cellular DNA damage response, because that miR-26b could target the mRNA of *ATM* and *Rb* genes via binding to their 3'UTRs, thus leading to decreased protein expression of ATM and Rb. In conclusion, our results indicate that E2F1 promotes the chemosensitization to cisplatin in ESCC. The effect may be due to the upregulation of miR-26b because cisplatin-induced cycle arrest depends on miR-26b, which may also disturb the DNA damage response by reducing the expression of ATM and Rb.

## Introduction

Esophageal squamous cell carcinoma (ESCC) is the main cause of death in patients with tumors, and its incidence is increasing annually in China 1. Clinical treatment of esophageal cancer is a challenging problem and is gaining increasing attention. In general, patients with an early TNM stage are mainly treated by surgery, while patients with a late stage are often treated with radiotherapy and/or chemotherapy in ESCC 2. However, patients with sensitivity to chemotherapy are relatively rare, and their 5-year survival rate is very poor in ESCC 1, 3. Moreover, targeted drugs based on EGFR, VEGF, PI3K and other pathways, which have been widely used in many cancers such as lung cancer and breast cancer, are not sufficiently effective in ESCC 4, 5. Therefore, it is important to investigate the mechanism of increasing the sensitivity of ESCC to chemotherapy.

E2F1, as a member of the E2F family, is an essential protein of the Rb/E2F1 signaling pathway. E2F1 regulates numerous genes, including those that encode S-phase-related proteins and miRNAs, which are involved in many cellular biological behaviors, including DNA synthesis and replication, mitotic checkpoint control, DNA damage repair, cell self-renewal, and differentiation 6, 7. The Rb/E2F1 pathway is often disrupted in many human tumors, in which the abnormal release of E2F1 induces transcriptional activation of its target genes and leads to cellular abnormalities 8, 9. Additionally, E2F1 expression was increased in ESCC cells after cisplatin treatment in our previous study 6. The upregulated expression of E2F1 may involve the treatment course of cisplatin, but its mechanism has not been elucidated in ESCC. miRNAs, as important non-coding RNAs that are involved in many biological processes in cells, could act oncogenes or tumor suppressor genes to regulate numerous related genes. Many studies have suggested the closely regulated relationship between E2F1 and miRNAs. E2F1 could directly regulate miRNAs, which further regulate other genes via binding to the 3`UTR regions. In our previous study, E2F1 involved the DNA damage response pathway, and miRNAs may participate in the process after cisplatin treatment, but its mechanism in ESCC has not been completely elucidated 6.

In our study, we investigated the potential mechanism involving the E2F1 and miRNA pathways to increase the sensitivity of ESCC to cisplatin. We found that E2F1 enhances the sensitivity of ESCC cells to cisplatin by regulating miR-26b and the cellular DNA damage response.

## Materials and methods

### Cell lines, Drug and Tissue specimens

Three human esophageal cancer cell lines (EC109, KYSE450 and EC9706) were purchased from the Cell Bank of Chinese Academy of Sciences (Shanghai, China). These cell lines were cultured in RPMI Medium 1640 with 10% fetal bovine serum (FBS) in a humidified incubator (5% CO_2_). Cisplatin was obtained from Sigma-Aldrich (St. Louis, MO).

Paired tumor tissues of ESCC and adjacent normal esophageal tissue were from surgical patients at the First Affiliated Hospital of Army Medical University. These tissues were cut into several pieces (~100 mg/piece), placed into freezing tubes and stored in liquid nitrogen immediately. Our study was approved by the Ethics Committee of the Army Medical University. All recruited patients in our study were given informed consent.

### Vector construction

The E2F1 expression plasmid 408 pSG5L-HA-E2F1 was obtained from Addgene (#10736) 10. Luciferase assays were performed using the pGL3-miR-26b-promoter vector containing the 26-bp DNA sequence of the miR-26b gene promoter region. The 26-bp DNA sequence was introduced into the Xho I and Hind III sites and cloned into the pGL3-basic vector (Promega, Madison, WI). The pGL3-miR-26b-promoter-MUT vector was generated by site-directed mutagenesis though overlap extension PCR 11. The primer sequences are listed in Table S1.

The pMIR-RB-3UTR-WT and pMIR-ATM-3UTR-WT plasmids contained the 3`UTR segment of the RB gene (NM_000321.2) and ATM gene (NM_000051), respectively, as well as the putative miR-26b binding site. The DNA segment was introduced into the MluI and HindIII sites and cloned into the pMIR-REPORT vector (Applied Biosystems, Foster City, CA, USA). The pMIR-Rb-3UTR-MUT and pMIR-ATM-3UTR-MUT vectors were the mutated seed regions of the miR-26b of pMIR-RB-3UTR-WT and pMIR-ATM-3UTR-WT vectors, respectively. The sequences were obtained by direct synthesis and are shown in Table S1.

### RNA oligoribonucleotides and transfection

The miR-26b mimics, miR-26b inhibitors, small interfering RNA (siRNA) against E2F1, and matched controls were obtained from Shanghai GenePharma Company. Cell transfection (vectors, siRNA, has-miR-26b mimics and inhibitors) were carried out by Lipofectamine 2000 (Invitrogen, Carlsbad, CA, USA). The sequences of the transfection assay are shown in Table S2.

### Quantitative real-time PCR (qRT-PCR)

Total RNA was extracted by RNAiso reagent (Takara, Dalian, China) from tissues (100 mg) and cultured cells (1× 10^6^). For quantitative detection of mature miR-26b, the All-in One^TM^ miRNA qRT-PCR Detection Kit of GeneCopoeia Company was used according to the manufacturer's instructions. qRT-PCR was performed using the Bio-Rad CFX Connect Real-Time PCR Detection System. The expression of miR-26b was normalized to that of U6 using the 2^-∆∆CT^ method for quantification. Related primers are shown in **Table S3.**

### Chromatin immunoprecipitation (ChIP)

The ChIP assay in EC109 cells was carried out using the EZ ChIP Kit (Upstate) according to the manufacturer's instructions. Approximately 1×10^7^ cells were harvested for each immunoprecipitation. The cross-linked chromatin was digested by micrococcal nuclease and sonicated to obtain DNA fragments with lengths of approximately 150-900 bp. Chromatin Immunoprecipitation was performed using an anti-E2F1 antibody and negative control normal IgG overnight. Eluotropic DNA was purified using spin columns. Finally, qRT-PCR was performed to detect the signal of candidate binding sites of E2F1 (Sites 1, 2, 3 and 4). The primers of ChIP assay are shown in Table S3.

### Reporter assay

For the reporter assay to detect the binding site of E2F1 in the promoter regions of miR-26b, the pGL3-miR-26b-promoter-WT or pGL3-miR-26b-promoter-MUT vector was mixed with the E2F1 overexpression plasmid (408 pSG5L HA E2F1) and pRL-TK, followed by co-transfection using Lipofectamine 2000. For the reporter assay to detect the binding site of miR-26b in the 3`UTR region of RB and ATM, the pMIR vector (pMIR-RB-3UTR-WT/MUT, pMIR-ATM-3UTR-WT/MUT) was mixed with mimics (miR-26b or negative control) and pRL-Tk, followed by co-transfection using Lipofectamine 2000 according to the manufacturer's instructions. Triplicate assays were performed for each independent group. The luciferase activities of Firefly and Renilla were detected by the Dual-Luciferase Reporter Assay System of Promega after transfection for 24 h.

### Cell cycle assay

Cell cycle analysis was performed in EC109 cells. Approximately 3× 10^5^ cells/well were seeded into 6-well plates. The miR-26b inhibitor or negative control was transfected the next day. Cisplatin treatment was carried out after transfection for 24 h. After treatment for 48 h, the collected cells were fixed in 70% ethanol overnight at 4 °C. Next, the fixed cells were stained with propidium iodide (PI) (Beyotime, Shanghai, China) for 30 min at 37 °C and then were analyzed by flow cytometry (BD Biosciences).

### Cell viability analysis

Approximately 8,000 cells/well were seeded on 96-well plates. The miR-26b mimics or negative controls were transfected the next day. Cell viability was measured after 12 h with or without cisplatin treatment using Cell Counting Kit-8 (Biyuntian, China) every 12 or 24 h. The viable cell number was quantified by the absorbance at a 450-nm wavelength.

### Lentiviral constructs and Nude mouse xenograft model

The lentiviruses (Si-E2F1 and negative control) were obtained from Shanghai GenePharma Company. The stably transfected EC109 cells were screened with puromycin after virus infection. The collected 2 × 10^6^ cells (EC109-NC, EC109-siE2F1) were mixed in 50% Matrigel (BD Biosciences) and were injected subcutaneously into approximately 4-week-old nude BALB/c mice. One week later, the mice started cisplatin (4 mg/kg) or equivoluminal physiological saline treatment (intraperitoneal injection, once a week, for 4 weeks) when the tumors were palpable 12. The tumor size was measured every three days, its volume was calculated as (length/2) × (width^2^), and the body weight of mice was monitored throughout the experiments. The experiment was performed in accordance with the Animal Research Committee Guidelines of Army Medical University.

### Western blotting

Total protein was extracted by RIPA Lysis Buffer (Beyotime Institute of Biotechnology) from cells or tissues. The supernatant was collected after centrifugation at 12,000 × g at 4°C for 5 min, and extracts with equal amounts of protein were subjected to SDS-PAGE and electrophoretic transfer onto polyvinylidene difluoride membranes. Next, the membranes were blocked with 5% nonfat dry milk for 2.5 h at room temperature. The membranes were then incubated with the following primary antibodies at 4°C overnight: anti-ATM (Epitomics; 1549-1), anti-RB (CST; 9309), anti-E2F1 (Upstate; 05-379), anti-GAPDH (KC5G5; Kangchen Bio-Tech). Finally, the membranes were incubated with a horseradish peroxidase-conjugated secondary antibody, and the proteins were visualized using the ECL Detection System (Thermo Scientific).

### Statistics

The data were recorded as means ± standard error. Each independent experiment was performed at least three times. Student's t-test was used to detect differences between two groups, and analysis of variance (ANOVA) was used to detect differences when the number of groups was greater than two. Multigroup comparisons were carried out by ANOVA with post-hoc contrasts using the Student-Newman-Keuls test. All differences were considered statistically significant when p < 0.05.

## Results

### Cisplatin induces the upregulation of E2F1 and miR-26b

Our previous study showed that cisplatin stably induces the expression of E2F1 protein, which then transcriptionally activates the expression of multiple genes that promote cell cycle progression and apoptosis. We also observed that EC109 and Kyse450 cells show stabilization and accumulation of E2F1 protein after treatment with cisplatin (10 μM for EC109 and 2 μM for Kyse450) at 12 h 6.

**Figure 1:**
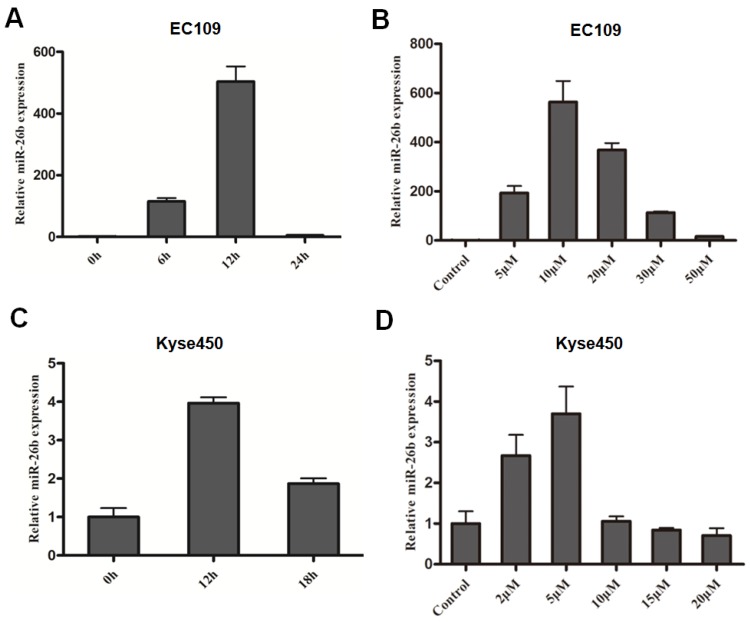
** miR-26b expression of cisplatin-treated ESCC cells.** Time-dependent(A) and dose-dependent (B) effect of miR-26b expression in cisplatin-treated EC109 cells. Time-dependent (C) and dose-dependent (D) effect of miR-26b expression with cisplatin in Kyse450 cells. The expression levels of miR-26b were measured by qRT-PCR.

In this study, the expression of miR-26b was consistent with the change of E2F1 expression. The expression of miR-26b in EC109 cells was increased approximately 20 fold at 6 hours and peaked at approximately 12 hours (approximately 150 fold) after treatment with 10 μM cisplatin (Figure 1A). For the concentration gradient effects of cisplatin, the expression of miR-26b increased at 5 μM cisplatin and peaked at 10 μM cisplatin. Next, as the concentration increased from 20 μM and 30 μM to 50 μM cisplatin, the expression of miR-26b was gradually decreased (Figure 1B). In the Kyse450 cells, the expression pattern of miR-26b was similar to that in EC109 cells. As shown in Figure 1C, the expression level of miR-26b was the highest after cisplatin treatment for 12 h (~3-fold increase) and then decreased at 18 h. For the concentration gradient effect of cisplatin, the expression of miR-26b increased at 2 μM cisplatin and peaked at 5 μM cisplatin (Figure 1D).

### Upregulation of miR-26b by cisplatin depends on the expression of E2F1

To investigate the role of E2F1 in miR-26b expression after cisplatin treatment, overexpression and loss-of-function assays of E2F1 were performed in both EC109 and Kyse450 cells.

**Figure 2:**
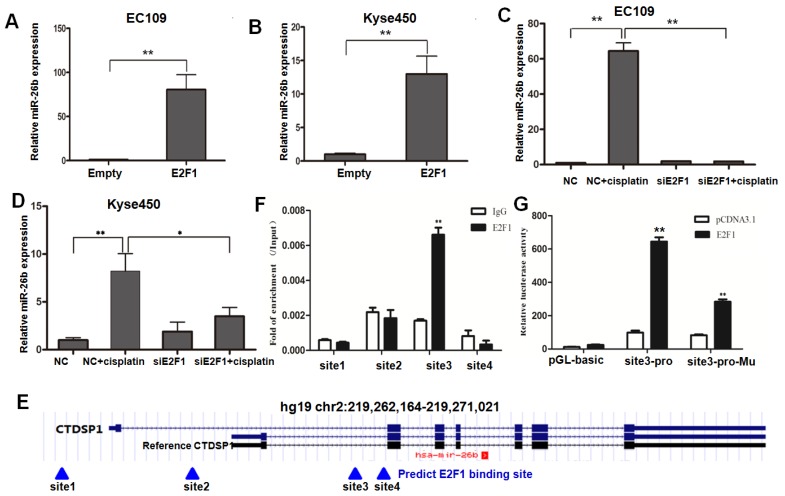
** Regulatory mechanism of miR-26b by E2F1. A and B.** The expression levels of miR-26b were measured by qRT-PCR in EC109 and Kyse450 cells for E2F1 overexpression, respectively.** C and D.** The expression levels of miR-26b were measured by qRT-PCR in both EC109 and Kyse450 cells for siRNA interference of E2F1 with or without cisplatin treatment.** E.** Bioinformatics predicted four potential E2F1 binding sites (sites 1 to 4) using the ChIP Mapper website. The miR-26b gene located in the intron region of CTDSP1. **F.** ChIP qRT-PCR of the potential binding sites in EC-109 cells. **G.** Luciferase reporter gene assay showed that pGL3 containing the site 3 fragment significantly increased luciferase activity when E2F1 was overexpressed. (*p<0.5, ** p<0.01).

On the one hand, overexpressed E2F1 significantly increased the expression level of miR-26b compared with the control group in both EC109 and Kyse450 cells (Figure 2A and B). On the other hand, when EC109 and Kyse450 cells were transfected with E2F1/control siRNA and treated with 10 μM cisplatin for 12 h, the expression level of miR-26b was significantly induced after cisplatin treatment in the control cells. However, the induction effect was prevent in both EC109 and Kyse450 cells when the E2F1 siRNA disrupted the E2F1 mRNA (Figure 2C and D), which was almost completely disappeared in EC109 cells. These data indicated that E2F1 mediates the molecular events of up-regulated miR-26b after cisplatin treatment.

### E2F1 activates the transcriptional activity of the miR-26b gene promoter

Having demonstrated that miR-26b was induced by E2F1, we next to investigate if miR-26b is a direct transcriptional target of E2F1. The miR-26b gene is located in the intron region of the CTDSP1 gene. As shown in Figure 2E, bioinformatics predicted four potential E2F1 binding sites (sites 1 to 4) using the ChIP Mapper website. Two binding sites (sites 1 and 2) were found at the transcription initiation site upstream of the CTDSP1 reference gene, and two (sites 3 and 4) were downstream. The ChIP assay suggested that the enrichment signal at site 3 was significantly higher than that of IgG using the E2F1 antibody, while no significant difference was found at the other three sites (Figure 2F).

In reporter gene assays, the 26-bp DNA fragment containing site 3 that did not contain promoter activity was induced in the pGL3-basic luciferase reporter gene. In EC109 cells, the reporter gene containing site 3 fragment significantly increased the luciferase activity when E2F1 was overexpressed. However, the activity of the reporter gene was significantly decreased after mutation of site 3 **(Figure 2G)**. Thus, E2F1 may promote the transcriptional activity of miR-26b by binding to the site 3. These results suggested that E2F1 was recruited and directly bound to the promoter region of miR-26b, leading to the upregulated expression of miR-26b at the transcriptional level.

### miR-26b inhibits the G1/S phase transition, thus result in the inhibition of ESCC cell growth

**Figure 3:**
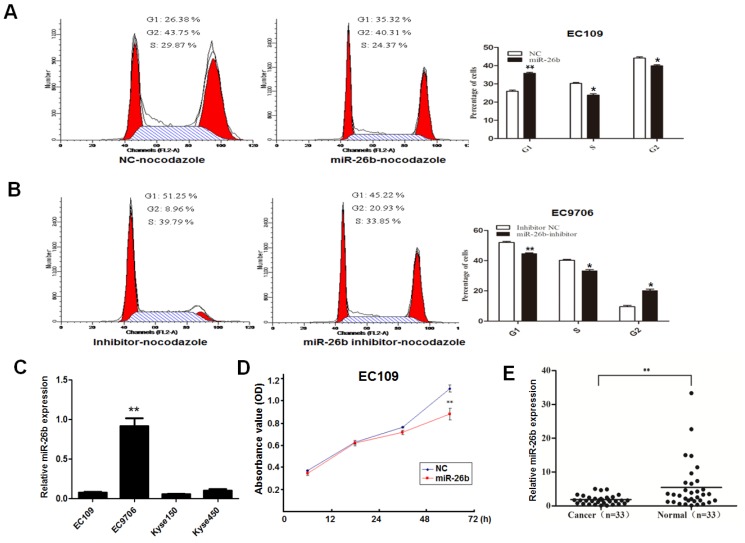
**Role of miR-26b in the growth and cell cycle distribution of ESCC cells.** EC109 cells (A) and EC9706 cells (B) were transfected with miR-26b mimics or miR-26b inhibitor, respectively. The 80ng/ml nocodazole was added 32 h after transfection, and the cells were cultured for an additional 16 h before harvesting for flow cytometry analysis. The percentages of cells in the G1, S, and G2 phases are shown**. C.** Basic expression of miR-26b was measured by qRT-PCR in EC109, EC9706, Kyse150 and Kyse450 cells.** D.** EC109 cells were transfection with miR-26b mimics or negative control (NC) control, and the cell viability was detected by CCK8 assay. **E.** The expression levels of miR-26b were measured by qRT-PCR in cancer tissues and adjacent normal tissues of 33 ESCC patients. (** p<0.01).

It has been elucidated that miR-26b is a downstream molecule of E2F1 above; we further explore its biological function. Overexpression or loss-of-function assays were carried out based on the baseline expression of miR-26b in different cell lines. Because the background expression of miR-26b was lowest in EC109 cells, the overexpression experiment was performed. For the effects of miR-26b on the cycle of EC109 cells, overexpression of miR-26b increased the number of G1 phase cells than controls (Figure 3A). By contrast, miR-26b background expression of EC9706 was highest in our collected ESCC cell lines, so loss-of-function assays were performed. As shown in Figure 3B, the number of G1 phase cells was significantly decreased when the expression of miR-26b was inhibited in EC9706 cells compared with the controls**.** These results suggested that miR-26b could inhibit the G1/S phase transition in ESCC cells. The expression patter of miR-26b in ESCC cell lines (EC109, EC9706, Kyse150 and Kyse 450) were showed in Figure 3C.

To further clarify the effect of miR-26b on cell growth, cell proliferation experiments were carried out in EC109 cells. We observed that cell viability was significantly reduced when EC109 cells were transfected with miR-26b mimics (Figure 3D). Notably, the expression of miR-26b was detected in 33 cases with ESCC. As shown in Figure 3E, the expression of miR-26b was significantly reduced in cancer tissues compared with that in paired normal esophageal tissues. Collectively, these data suggested that miR-26b has a significant inhibitory effect on the ESCC cell growth.

### Cisplatin-induced cycle arrest of ESCC depends on miR-26b

It is reported that cisplatin is important in regulating cell cycle arrest, but its mechanism is not fully clarified in ESCC; and several studies also suggested that miR-26b involves in cell cycle regulation. Thus, we further analyzed its potential effect on the cell cycle in EC109 cells with cisplatin treatment.

**Figure 4:**
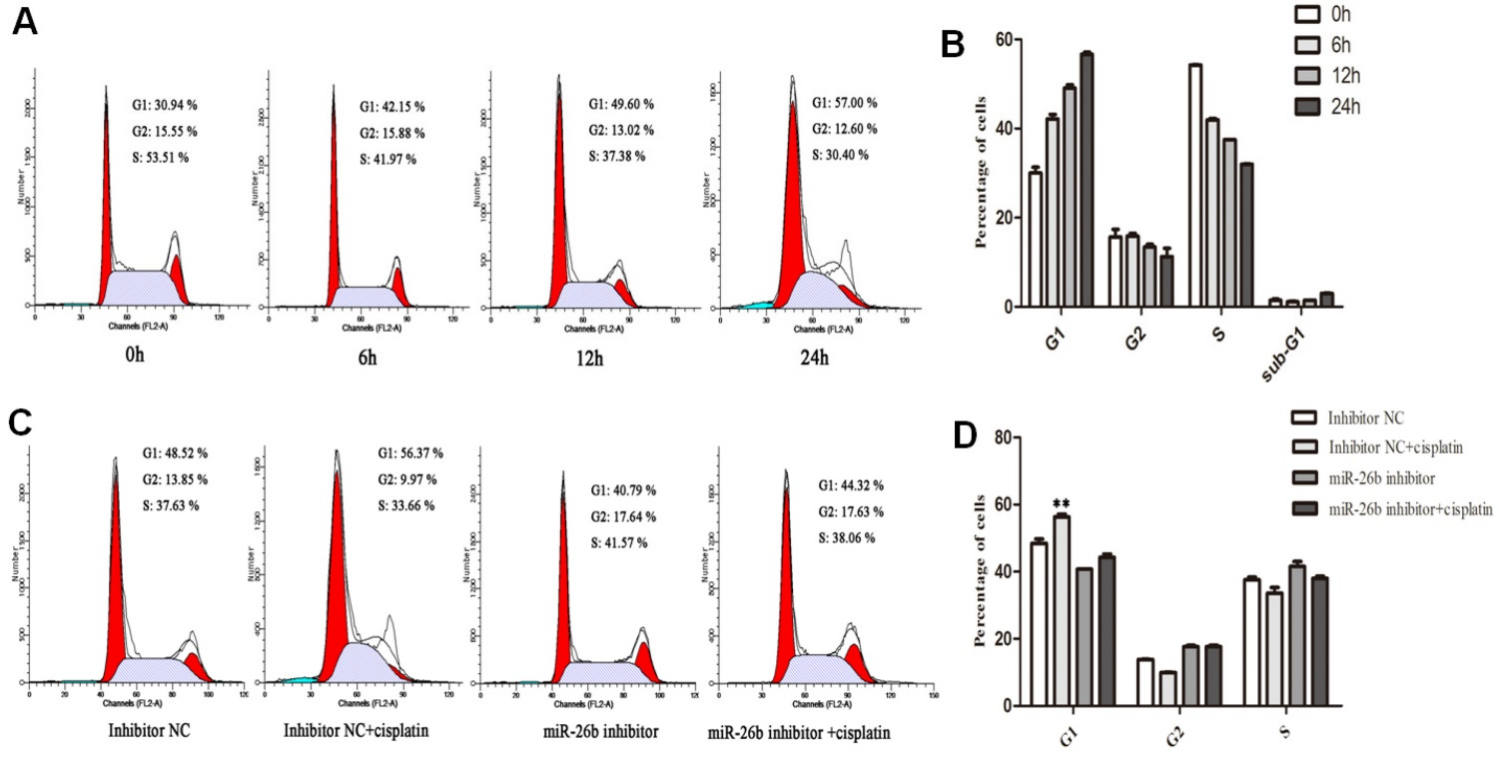
** Effect of miR-26b on the cell cycle distribution in EC109 cells with cisplatin treatment. A and B.** EC109 cells were treated by cisplatin for 6, 12 and 24 h ,and the percentages of cells in the G1, S, and G2 phases were analysis by flow cytometry. **C and D.** EC 109 cells were transfected with miR-26b inhibitor or inhibitor negative control (NC) for 24h, and the cells was added in cisplatin for an additional 12 h before harvesting for flow cytometry analysis. (** p<0.01).

As shown in Figure 4A and 4B, the proportion of G1 phase in the cell cycle increased, while the proportion of G2 and S phase decreased with time after 6, 12 and 24 h of cisplatin treatment in EC109 cells. These results suggested that cisplatin has a blocking effect on the G1/S phase of EC109 cells with time. Next, the miR-26b inhibitor was used to block miR-26b expression, and its effects on cisplatin-induced cell cycle arrest were analyzed. We observed that the proportion of G1 phase cells with cisplatin treatment was significantly decreased when miR-26b expression was blocked in EC109 cells (Figure 4C and 4D). These results suggested that the cisplatin-induced cycle arrest of ESCC depends on miR-26b *in vitro*.

### E2F1 enhances the chemosensitization of cisplatin in EC109 cells *in vivo*

**Figure 5:**
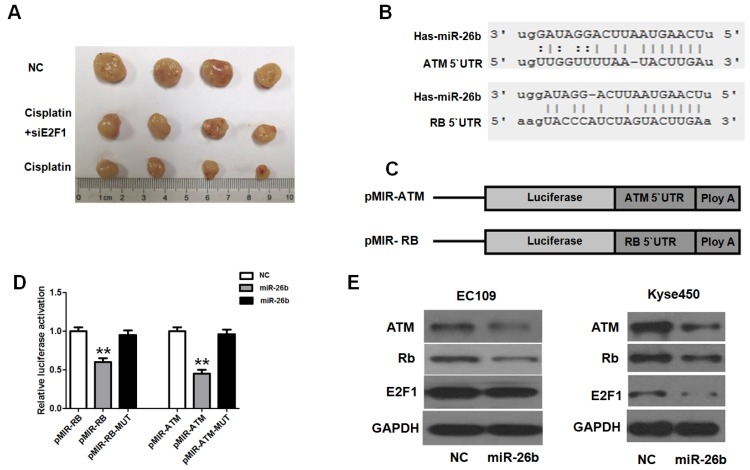
** Analysis of E2F1 regarding the chemosensitization to cisplatin *in vivo*.** A. Mice were injected subcutaneously with Si-E2F1 or negative control(NC) in EC109 cells, and received cisplatin treatment with equal dose (4 mg/kg). The final xenografts were separated and weighted. B. In silico analysis (TargetScan, miRanda and PicTar) revealed that miR-26b has a conserved binding site in the 3′UTR regions of ATM and Rb. C and D. Luciferase reporter assay indicated that miR-26b decreased the luciferase activity, but the luciferase activity almost rose to control levels when the binding sites were mutated. E. The reduced ATM, Rb, and E2F1 expression was analysis by Western blot in EC109 and Kyse450 cells transfected with NC or miR-26b mimics for 48 h. (** p<0.01).

To further investigate whether E2F1 could affect the chemosensitization of cisplatin *in vivo*, the cell model of Si-E2F1 EC109 and normal controls were constructed using lentiviruses. After 4 weeks of cisplatin treatment, as observed in Figure 5A, the tumor volumes of the cisplatin treatment group (cisplatin group and cisplatin with Si-E2F1 group) were significantly lower than those of the NC group. However, the tumor volumes of the cisplatin with Si-E2F1 group were higher than those of the cisplatin group. Thus, our study suggested that E2F1 may promote the chemosensitization of cisplatin in EC109 cells.

### miR-26b is activated by E2F1 targets ATM and Rb via binding to their 3'UTRs

miR-26b could be transcriptionally activated by E2F1, further regulating its target gene expression by binding to the 3'UTR of mRNA. In *in silico* analysis (TargetScan and PicTar), conserved binding sites of miR-26b in the 3′UTR region of ATM and Rb genes were found (Figure 5B). To verify the binding ability of these sites, reporter vector containing the 3′UTR regions of Rb or ATM were constructed. The luciferase reporter assay indicated that miR-26b decreased the luciferase activity, but the luciferase activity almost rose to control levels when the binding sites were mutated (Figure 5C and D). Moreover, protein expression was analyzed when miR-26b was overexpressed in EC109 and KYSE450 cells. In these two cell lines, ATM and Rb proteins were significantly decreased when miR-26b was over expressed (Figure 5E). Moreover, E2F1 expression was decreased in KYSE450 but was not altered significantly in EC109 cells. These results suggested that miR-26b could regulate the expression of ATM and Rb.

## Discussion

In our previous study, persistent expression of E2F1 was found in ESCC cells after cisplatin treatment 6. Here, we further identified that E2F1 directly binds to the promoter of the miR-26b gene, leading to the increased expression of miR-26b. Moreover, the expression of miR-26b was decreased in cancer tissues compared with that in normal esophagus tissues of patients with ESCC. The result was consistent with some previous findings in breast cancer 13, nasopharyngeal carcinoma 14, glioma 15, liver cancer 16, and colon cancer 17, indicating that lower miR-26b expression is a common phenomenon in various tumors and is closely related to tumorigenesis. Additionally, miR-26b could inhibit the proliferation of EC109 cells. These results suggested that miR-26b may be a tumor suppressor gene in ESCC and may serve as a potential therapeutic target.

We found that E2F1 increased the chemosensitization of cisplatin in EC109 cells in our present study. In addition, the cell viability of the cisplatin with siE2F1 group was significantly higher than that of the cisplatin group, indicating that the chemosensitization of cisplatin relies on the expression of E2F1. The concealed mechanism is complex, and we speculate that the effect may be due to the expression of miR-26b because the cisplatin-induced cycle arrest of ESCC depends on miR-26b. In ESCC cells, miR-26b plays important role in regulating G1/S arrest in the cell cycle, and miR-26b inhibition could inhibit the cisplatin-induced blockade of the G1/S phase. Consistently, some studies have suggested that miR-26b can target several G1/S phase-related genes such as CDK6, cyclinE1, CyclinE2, CyclinD2 and MYC 18, 19, 20, 21. Notably, miR-26b decreased the expression of Rb, and E2F1. Rb is the upstream regulator of E2F1 and determines the release of E2F1 through phosphorylation, and the downregulated expression of Rb may affect the function of the Rb/E2F1 pathway, further influencing the expression of miR-26b. These results suggested that E2F1 and miR-26b interactions in a feedback loop and regulate the G1/S phase transition in ESCC cells. So, E2F1 increased the chemosensitization of cisplatin likely through the G1/S arrest effect of miR-26b.

In addition, the increased chemosensitization of cisplatin by E2F1 may be due to the reduced DNA damage response though miR-26b. We found that ATM was direct target of miR-26b, and miR-26b decreased the expression of ATM in ESCC cells. It has been reported that ATM participates in the cisplatin-induced DNA damage response, and activation of ATM induces activation of cell cycle checkpoints and DNA repair responses 22, 23. Moreover, previous studies also found that E2F1 can enhances the ATM expression level through enhancing ATM promoter activity 24. After cisplatin treatment, multiple DNA repair mechanisms are usually activated, responding to cisplatin-damaged DNA. To a certain extent, these activated DNA repair systems decreased the cytotoxic efficacy of the chemotherapy drugs and is an important mechanism involving cisplatin resistance in cancer cells 25. The decreased ATM expression via miR-302 in E2F1-ATM pathways can lead to increased sensitivity of tumor cells to drugs like cisplatin 26. ATM deficiency sensitizes the cells to cisplatin-induced apoptosis 27. Therefore, we speculate that miR-26b participates in the regulation of the DNA damage pathway through abrogating ATM activation, finally affecting DNA repair and cell proliferation.

**Figure 6:**
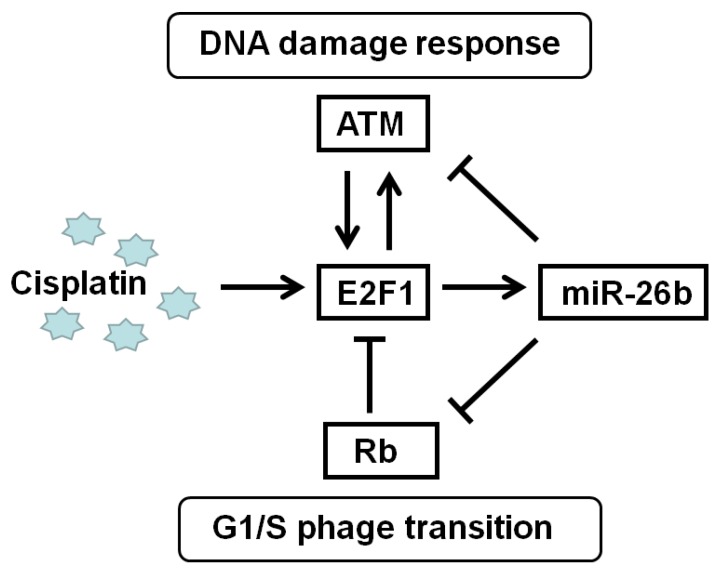
Regulation pattern of E2F1/miR-26b molecular network on cisplatin treatment.

The relationship among E2F1, Rb, and ATM is complex, but a feedback network is formed via miR-26b to participate in the treatment of ESCC with cisplatin. Deregulation of the Rb-E2F1 pathway often occurs in most cancers involving mutations or epigenetic events, cell cycle progression and apoptosis induction in response to DNA damage through its capacity to activate p53/p73 death pathways 26, 28. E2F1 activates ATM using a mechanism initiated by DNA damage, and E2F1 also directly enhances ATM through enhancing its promoter activity. E2F1 affects the ATM signaling pathway, which induces Chk2 and p53 phosphorylation and is involved in cell apoptosis 29. In response to DNA double-strand breaks, ATM could directly phosphorylate E2F1 on Ser31, resulting in E2F1 protein stabilization 30. In our study, miR-26b was the E2F1 target gene and participated in the Rb/E2F1-ATM pathway. miR-26b could decrease the mRNA expression of Rb and ATM via their UTR binding sites, suggesting that miR-26b may be an important regulator of the Rb/E2F1-ATM pathway in Figure 6.

Notably, the effect of E2F1 increased chemosensitization of cisplatin may be cell specific. Some studies suggested that E2F1 is associated with malignant phenotypes in some cancers 31. In human tumor cells, overexpressed E2F1 is involved in multidrug resistance; E2F1 increases the activity of the MDR1 promoter, resulted in higher P-gp levels 32. In our study, the chemosensitization of cisplatin relies on E2F1 in ESCC. However, its target gene miR-26b may negatively regulate E2F1 in part ESCC cell line such as Kyse450. These differences may be due to the cancer type and expression profile of cancer cells and need to be confirmed in further research. These results also suggested that the Rb/E2F1/miR-26 pathway should be differentially analyzed in different ESCC to evaluate possible drug targets.

In conclusion, E2F1 increases the chemosensitization of cisplatin in ESCC, and the effect may be due to the upregulation of its target gene miR-26b. miR-26b acts as a tumor suppressor gene to regulate cell cycle arrest, and cisplatin-induced cycle arrest depends on miR-26b in ESCC. Additionally, miR-26b may disturb the DNA damage response by reducing the expression of ATM.

## Supplementary Material

Supplementary figures and tables.

## References

[1] Chen W, Zheng R, Baade PD, Zhang S, Zeng H, Bray F (2016). Cancer statistics in China, 2015. CA Cancer J Clin.

[2] Kato H, Nakajima M (2013). Treatments for esophageal cancer: a review. Gen Thorac Cardiovasc Surg.

[3] Lordick F, Holscher AH, Haustermans K, Wittekind C (2013). Multimodal treatment of esophageal cancer. Langenbecks Arch Surg.

[4] Uzunoglu FG, Koenig AM, Izbicki JR (2014). The potential for targeting HER2 therapeutically in esophageal cancer - a grasp at straws?. Expert Opin Ther Targets.

[5] Lyons TG, Ku GY (2017). Systemic therapy for esophagogastric cancer: targeted therapies. Chin Clin Oncol.

[6] Zhang K, Dai L, Zhang B, Xu X, Shi J, Fu L (2015). miR-203 is a direct transcriptional target of E2F1 and causes G1 arrest in esophageal cancer cells. J Cell Physiol.

[7] Cam H, Dynlacht BD (2003). Emerging roles for E2F: beyond the G1/S transition and DNA replication. Cancer Cell.

[8] Chen HZ, Tsai SY, Leone G (2009). Emerging roles of E2Fs in cancer: an exit from cell cycle control. Nat Rev Cancer.

[9] Polager S, Ginsberg D (2009). p53 and E2f: partners in life and death. Nat Rev Cancer.

[10] Sellers WR, Novitch BG, Miyake S, Heith A, Otterson GA, Kaye FJ (1998). Stable binding to E2F is not required for the retinoblastoma protein to activate transcription, promote differentiation, and suppress tumor cell growth. Genes Dev.

[11] Bryksin AV, Matsumura I (2010). Overlap extension PCR cloning: a simple and reliable way to create recombinant plasmids. Biotechniques.

[12] Yu L, Gu C, Zhong D, Shi L, Kong Y, Zhou Z (2014). Induction of autophagy counteracts the anticancer effect of cisplatin in human esophageal cancer cells with acquired drug resistance. Cancer Lett.

[13] Liu XX, Li XJ, Zhang B, Liang YJ, Zhou CX, Cao DX (2011). MicroRNA-26b is underexpressed in human breast cancer and induces cell apoptosis by targeting SLC7A11. FEBS Lett.

[14] Ji Y, He Y, Liu L, Chong X (2010). MiRNA-26b regulates the expression of cyclooxygenase-2 in desferrioxamine-treated CNE cells. FEBS Lett.

[15] Wu N, Zhao X, Liu M, Liu H, Yao W, Zhang Y (2011). Role of microRNA-26b in glioma development and its mediated regulation on EphA2. PLoS One.

[16] Ji J, Shi J, Budhu A, Yu Z, Forgues M, Roessler S (2009). MicroRNA expression, survival, and response to interferon in liver cancer. N Engl J Med.

[17] Zhang C, Tong J, Huang G (2013). Nicotinamide phosphoribosyl transferase (Nampt) is a target of microRNA-26b in colorectal cancer cells. PLoS One.

[18] Zhu Y, Lu Y, Zhang Q, Liu JJ, Li TJ, Yang JR (2012). MicroRNA-26a/b and their host genes cooperate to inhibit the G1/S transition by activating the pRb protein. Nucleic Acids Res.

[19] Kota J, Chivukula RR, O'Donnell KA, Wentzel EA, Montgomery CL, Hwang HW (2009). Therapeutic microRNA delivery suppresses tumorigenesis in a murine liver cancer model. Cell.

[20] Leone G, DeGregori J, Sears R, Jakoi L, Nevins JR (1997). Myc and Ras collaborate in inducing accumulation of active cyclin E/Cdk2 and E2F. Nature.

[21] Matsumura I, Tanaka H, Kanakura Y (2003). E2F1 and c-Myc in cell growth and death. Cell Cycle.

[22] Colton SL, Xu XS, Wang YA, Wang G (2006). The involvement of ataxia-telangiectasia mutated protein activation in nucleotide excision repair-facilitated cell survival with cisplatin treatment. J Biol Chem.

[23] Bensimon A, Aebersold R, Shiloh Y (2011). Beyond ATM: the protein kinase landscape of the DNA damage response. FEBS Lett.

[24] Berkovich E, Ginsberg D (2003). ATM is a target for positive regulation by E2F-1. Oncogene.

[25] Kothandapani A, Dangeti VS, Brown AR, Banze LA, Wang XH, Sobol RW (2011). Novel role of base excision repair in mediating cisplatin cytotoxicity. J Biol Chem.

[26] Cataldo A, Cheung DG, Balsari A, Tagliabue E, Coppola V, Iorio MV (2016). miR-302b enhances breast cancer cell sensitivity to cisplatin by regulating E2F1 and the cellular DNA damage response. Oncotarget.

[27] Wang J, Pabla N, Wang CY, Wang W, Schoenlein PV, Dong Z (2006). Caspase-mediated cleavage of ATM during cisplatin-induced tubular cell apoptosis: inactivation of its kinase activity toward p53. Am J Physiol Renal Physiol.

[28] Kowalik TF, DeGregori J, Leone G, Jakoi L, Nevins JR (1998). E2F1-specific induction of apoptosis and p53 accumulation, which is blocked by Mdm2. Cell Growth Differ.

[29] Powers JT, Hong S, Mayhew CN, Rogers PM, Knudsen ES, Johnson DG (2004). E2F1 uses the ATM signaling pathway to induce p53 and Chk2 phosphorylation and apoptosis. Mol Cancer Res.

[30] Lin WC, Lin FT, Nevins JR (2001). Selective induction of E2F1 in response to DNA damage, mediated by ATM-dependent phosphorylation. Genes Dev.

[31] Han S, Park K, Bae BN, Kim KH, Kim HJ, Kim YD (2003). E2F1 expression is related with the poor survival of lymph node-positive breast cancer patients treated with fluorouracil, doxorubicin and cyclophosphamide. Breast Cancer Res Treat.

[32] Yan LH, Wei WY, Cao WL, Zhang XS, Xie YB, Xiao Q (2014). Overexpression of E2F1 in human gastric carcinoma is involved in anti-cancer drug resistance. BMC Cancer.

