# Supplementary material for: E2F1 promotes cancer cell sensitivity to cisplatin by regulating the cellular DNA damage response through miR-26b in esophageal squamous cell carcinoma
Source: J Cancer. 2020 Jan 1;11(2):301–10. doi: 10.7150/jca.33983 (PMC6930434; doi:10.7150/jca.33983)
Supplement: Supplementary file 1 — Supplementary figures and tables. [file jcav11p0301s1.pdf]

Table S1. Primers for vector construction of the reporter pGL3 or pMIR-REPORT

Name	Sequence (5'-3')
pGL3-miR-26b-promoter	Forward primer:<br>CCGCTCGAG CGGCGACCGTCCCATTCAAG
	Revere primer:<br>CCCAAGCTTCCTTGCTGATCTGAGTAATGACGG
pGL3-miR-26b-promoter -MUT	Forward primer:<br>ATGTTTGCATCCGCCTCGTTGGTAGGAAACTCCATGTTG
	Revere primer:<br>CAACATGGAGTTTCCTACCAACGAGGCGGATGCAAACAT
pMIR-ATM-3UTR-WT	CTATTGTGGGTTTTTTTGAATGTTGGTTTTAATACTTGATTTAATCACCA<br>CTCAAAAATG
pMIR-ATM-3UTR-MU	CTATTGTGGGTTTTTTTGAATGTaGGTTaTttatgaactTTTAATCACCA<br>CTCAAAAATG
pMIR-RB-3UTR-WT	CATTCAGATCACTGAATTTATAAAGTACCCATCTAGTACTTGAAAAAGTA<br>AAGTGTTCTGCCAGAT
pMIR-RB-3UTR-MU	CATTCAGATCACTGAATTTATAAAGatCggAaCTtGatgaactAAAAGTA<br>AAGTGTTCTGCCAGAT

Table S2 Sequences of the transfection assay

siE2F1	GACCACCUGAUGAAUAUCUTT
	AGAUAUUCAUCAGGUGGUCTT
NC	UUCUCCGAACGUGUCACGUTT
	ACGUGACACGUUCGGAGAATT
miR-26b mimics	UUCAAGUAAUUCAGGAUAGGU
	CCUGUUCUCCAUUACUUGGCUC
inhibitor NC	CAGUACUUUUGUGUAGUACAA
anti-miR-26b	ACCUAUCCUGAAUUACUUGAA

Table S3. Primers for Real-time PCR and ChIP-PCR

Target Name	Target sequence	Products
Site 1	F: TCCAACTTGTGCCTCCCTAC	227bp
	R: GCCCTGAAGACTGAGTCCAG	
Site 2	F: GGCAAGGTGGAAAGGCTAGT	193bp
	R: GGGTACGAAGTCCTCACTGC	
Site 3	F: GTCCCATTCAAGAGCGTGAT	166bp
	R: TTTCCTCGTTGAGGACTTGG	
Site 4	F: AAACCCGGCCTGATTAAAGT	211bp
	R: CCTGACGGAGAAAGAAGTGC	
